# Significant Luminescence Enhancement of Ga-Doped WS_2_ Monolayers Grown by CVD

**DOI:** 10.1021/acsomega.5c01066

**Published:** 2025-04-09

**Authors:** Shuai Zhang, Andre N. Barbosa, Munique Eva Paiva de Araujo Monteiro de Barros, Alexandre Mello, Kevin Lizárraga, Pedro Paulo de Mello Venezuela, Fernando Lázaro Freire

**Affiliations:** † Department of Physics, Pontifícia Universidade Católica do Rio de Janeiro, Rio de Janeiro 22451-900, Brazil; ‡ Laboratory of Surfaces and Nanostructures, Brazilian Center for Physics Research, Rio de Janeiro 22290-180, Brazil; § Institute of Physics, Fluminense Federal University, Niteroi 24210-346, Brazil; ∥ Departamento de Ciencias, Sección Física, Pontificia Universidad Católica del Perú, Av. Universitaria 1801, Lima 32, Peru

## Abstract

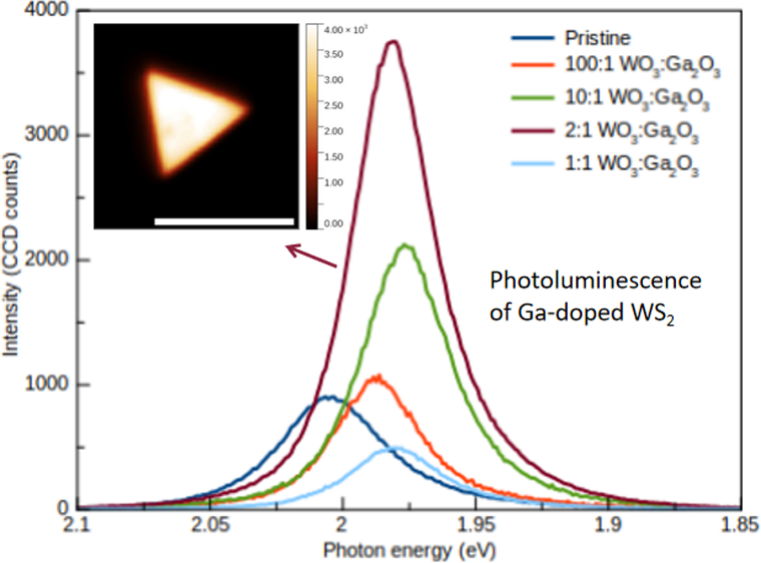

Monolayer tungsten disulfide (WS_2_) is a direct-band-gap semiconductor that has excellent luminescence properties, which are of great interest for optoelectronic applications. In this study, we investigated the effect of gallium (Ga) on WS_2_ monolayers grown by chemical vapor deposition. Our results indicate that Ga-doped WS_2_ exhibits a 3.6-fold increase in photoluminescence intensity for doped samples compared to pristine WS_2_. To confirm the existence of Ga in the WS_2_ structures, resonance Raman spectroscopy and X-ray photoelectron spectroscopy (XPS) were utilized as characterization methods. A redshift of the XPS spectrum was observed as well as an increase in the disorder-related Raman modes, which were attributed to the influence of Ga. XPS analysis and ab initio electronic structure calculations reveal the presence of substitutional Ga atoms as well as Ga atoms adsorbed on WS_2_ surfaces.

## 1 Introduction

Transition-metal dichalcogenides (TMDs) are a class of 2D materials that have garnered significant interest due to their unique physicochemical and photoelectronic properties.^1−3^ This makes them highly promising candidates for photoelectronic applications.^4−9^ Single-layer TMDs feature a three-plane structure where the metal (Me) atomic plane is sandwiched by two chalcogen (X) atomic planes via a strong ion–covalent bond, forming an X–Me–X structure.^10^ Unlike bulk or multilayer TMDs, many monolayer TMDs possess a direct band gap.^11,12^ One such material is single-layer WS_2_, which is becoming increasingly popular due to its appropriate band gap (1.9 eV),^13,14^ strong spin–orbit coupling, and high PL quantum yield.^15−17^ These properties give rise to the strong luminescence intensity in WS_2_ monolayers, which stand out from those of other TMDs. In fact, as previously mentioned, single-layer WS_2_ has a significantly higher PL quantum yield, of around 6%, when compared to other 2D materials (which is about 0.1% for MoS_2_, for instance).^17^

By strategically incorporating and controlling impurities or defects within the lattice of WS_2_, one can significantly alter a specific property to improve the performance of devices for a target application, such as photonics or catalysis. Additionally, defects in TMD structures can impact the PL intensity, as well as other physical or chemical factors, such as the adsorption of complex molecules on the basal plane of monolayered crystals,^18^ which is paramount in catalysis applications. In WS_2_, defects usually include W vacancies and S vacancies.^19−21^ However, defects also provide an opportunity for doping.^22,23^ By incorporating different atoms into a semiconductor, it is possible to tailor its photoelectronic properties by controlling dopants in the material. For instance, carbon, niobium, and tantalum can be incorporated into TMDs to obtain p-type electronic properties.^24−26^ In a recent study, gallium-doped MoS_2_ monolayers showed 2 orders of magnitude enhancement in their photoluminescence (PL) response.^22^ Plasma surface treatment is another efficient way to dope TMDs with atoms. It has been shown that, through cold plasma treatment, nitrogen was incorporated into the lattice and improved the luminescence properties of WS_2_ monolayers.^27^ An extensive discussion on the potential applications of monolayers WS_2_ can be found in a recent review.^28^

Aiming toward application in photonic and electronic devices, a significant effort has been made to synthesize wafer-sized TMD samples, making them compatible with current technology.^29−31^ Although good-quality single-crystal WS_2_ can be isolated through an exfoliation method, it is unsuitable for high-volume industrial fabrication. Due to the low cost and high yield, chemical vapor deposition (CVD) and atomic layer deposition are considered the most promising growth strategies to obtain high-quality and large-area 2D TMDs and heterostructures since other synthesis methods, such as epitaxial growth or other chemical routes, either lack scalability or yield irregular samples.^32−35^ CVD allows for efficient TMD growth using a variety of precursor forms, including powders or/and liquids. Sometimes, salt, like NaCl or KBr, is used to generate a transition-metal halide with a reduced melting point, as compared to their transition-metal oxide counterparts, and generate a vapor pressure necessary for deposition to occur on the target substrate. To substitutionally dope TMDs with transition metals, the dopant precursor is either directly mixed with the host transition-metal precursors, placed upstream, or placed onto the target substrate. The approximate dopant concentration is determined by the ratio of the host and the dopant metal precursor. Using this technique, elements from group V (vanadium, niobium, tantalum), rhenium, iron, tin, and lanthanides such as cerium, erbium, and ytterbium have been substitutionally doped in TMDs.^28^ However, when it comes to doped TMDs, they are typically produced by creating defects in the crystalline structure, which can weaken the final properties. For that reason, it is essential to develop, or improve, an in situ doping method that can avoid structural damage while effectively introducing dopants into TMDs.

The in situ doping strategy has many advantages over other impurity incorporation strategies since it is a one-step process rather than a multiple-step process. Also, the preparation of the precursors is rather simple, independent of a dry, powder-based mix method^36^ or wet-method-based CVD processes. Other surface treatment techniques that are employed, such as ion bombardment, plasma treatment, or dip-coating, also require further steps in the preparation of the samples.^37−39^ These methods require further steps that hinder the final yield and scalability of the CVD process and the implementation of TMD-based devices in the industry. In situ-based growth was employed in previous works by some of the authors, as well as other research groups targeting the modification of different properties, such as the magnetic response of vanadium-doped WSe_2_,^40^ as well as luminescence properties in V-WSe_2_ monolayers.^41^

This study introduces an in situ CVD approach for synthesizing WS_2_ while simultaneously doping it with gallium (Ga) atoms. The inspiration for doping WS_2_ with Ga arises from the research conducted by B. Liu and colleagues, where they notably improved the PL intensity of MoS_2_ through Ga doping.^22^ The Ga-doped samples were synthesized by mixing WO_3_ and Ga_2_O_3_ powders in different mass ratios (*m*(WO_3_):*m*(Ga_2_O_3_) of 100:1, 10:1, 2:1, and 1:1), and their PL intensities were compared with that of pristine WS_2_ monolayers. The PL intensity shows a dependence on the precursors’ mass ratio. When the amount of Ga dopant increases from a mass ratio of 100:1 to 1:1, the PL intensity initially increases, reaching a maximum at a mass ratio of 2:1, i.e., up to 3.6 times the PL intensity of pristine samples. It was followed by a decrease for Ga-richer precursor mixtures. In the case of the ratio 1:1, the PL intensity is weaker than that observed for pristine samples. The adsorption of Ga atoms in substitutional W sites has been revealed by X-ray photoelectron spectroscopy (XPS) measurements and confirmed by ab initio calculations.

## 2 Synthesis, Characterization, and Theoretical Procedures

The synthesis process followed a similar protocol developed in ref (37). The details of the growth process can be found in the Supporting Information (SM). WS_2_ was prepared by CVD. Sulfur (S) and tungsten trioxide (WO_3_) were used as precursors for the preparation of WS_2_, while doped WS_2_ mixtures of WO_3_ and gallium trioxide (Ga_2_O_3_) were used with different mass ratios. In this study, the mass ratios of the mixture were *m*(WO_3_):*m*(Ga_2_O_3_) = 100:1, 10:1, 2:1, and 1:1. 20 mg of WS_2_ powder or powder mixed with Ga_2_O_3_ was put in a crucible. A SiO_2_ (275 nm thick)/Si wafer was placed diagonally above the powder, with one edge of the wafer placed at the bottom of the crucible and the opposite edge placed on the inside wall of the crucible (see the SM). In addition, 300 mg of sulfur powder was positioned in another crucible. The amount of S was the same for the growth of the pristine and doped samples. The two crucibles were placed into a quartz tube, and it was placed in a two-stage furnace. WS_2_ monolayers were synthesized on the SiO_2_ surface. The experimental scheme and the temperature curve profile are shown in Figure S1.

Atomic force microscopy (AFM), Raman, and PL measurements were performed by using the same instrument (NT-MDT NTEGRA SPECTRA), equipped with a solid-state 532 nm laser. The diameter of the focused laser spot is about 1 micrometer, and its power ranges from 0.02 to 2 mW, controlled by a variable neutral density filter. The focal length of the spectrometer is 52 cm. Raman spectra were taken using an 1800 L/mm grating, yielding a resolution of 0.8 cm^–1^. PL maps were performed using a 150 L/mm grating, yielding a 4 meV resolution. The PL map intensity data reported in the text are related to the average data acquired from several maps performed in different samples to ascertain the reproducibility of the results. Also, the 2LA(M)+E_2g_ mode was used as a normalizing parameter for the PL peaks and maintained at a fixed value (∼100 counts) during the measurements.

The surface analysis chamber is equipped with a SPECS PHOIBOS 100/150 hemispherical analyzer. XPS measurements were conducted at a pressure of 1.2 × 10^–9^ Torr, and the Al-Kα X-ray line was used. During measurement, the analyzer was positioned at a 90 ° angle to the sample surface normal. XPS data analysis was performed using CasaXPS software. The C1s peak was used for XPS data energy calibration. The measured C–C peak position was set to 284.6 eV, and this calibration was used for all data from the same sample. The XPS line was deconvolved using pseudo-Voigt line shapes, and the Shirley background was used as the baseline.

Density functional theory (DFT) calculations were performed using the VASP code.^37^ For the exchange–correlation functional, the Perdew–Burke–Ernzerhof generalized gradient approximation was used, including Tkatchenko–Scheffler dispersion corrections.^38^ Periodic boundary conditions with 4 × 4 × 1 and 5 × 5 × 1 supercells were considered. A cutoff energy of 500 eV and a 3 × 3 × 1 grid of *k*-points were used. The formation energies (*E*_F_) were calculated using the approach described in ref (39).

## 3 Results and Discussions

**Figure 1:**
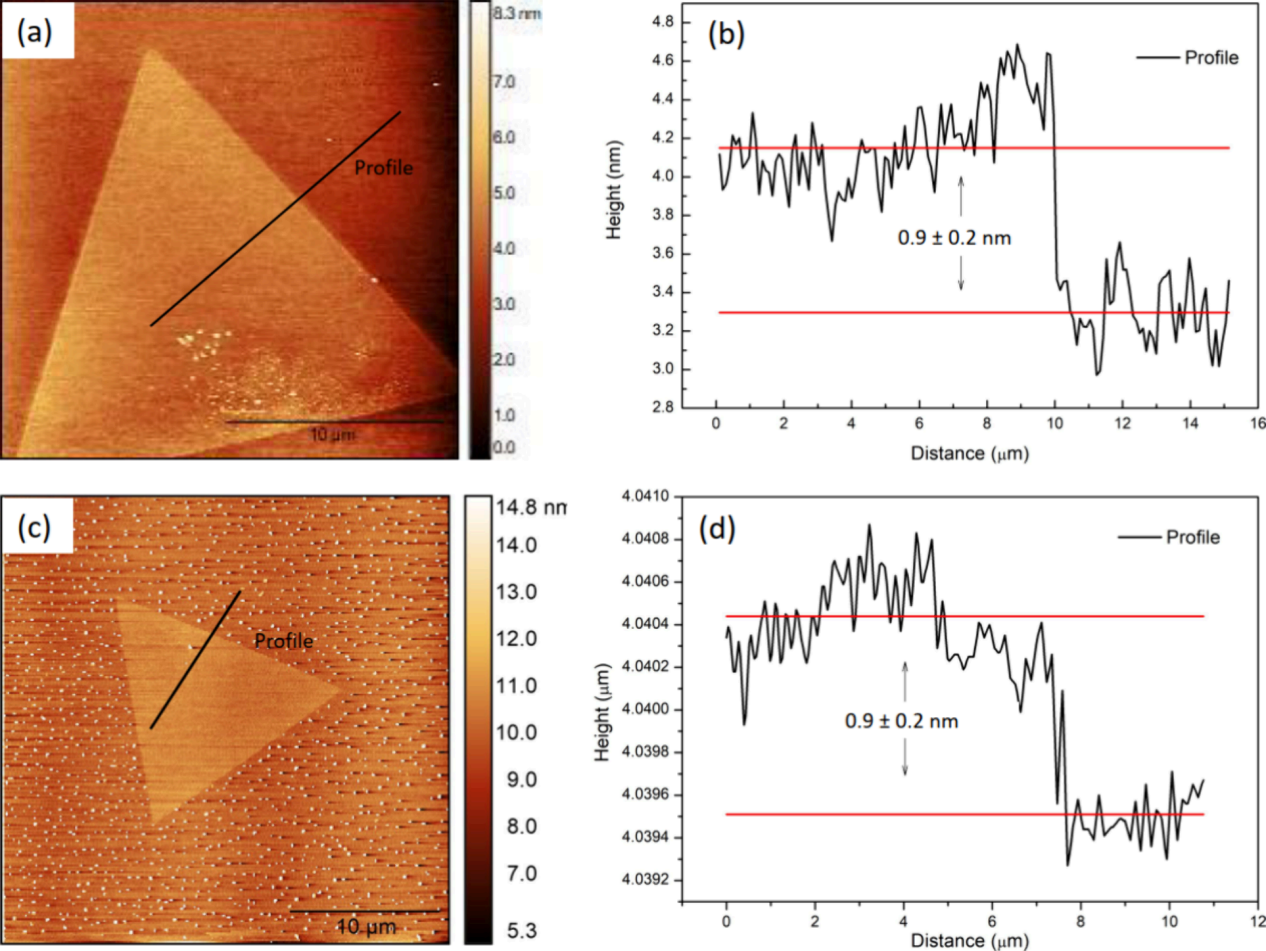
(a) and (b) AFM image and height profile of the pristine monolayer WS_2_, whose height is about 0.9 ± 0.2 nm; (c) and (d) AFM image and height profile of the 2:1 Ga-doped WS_2_ monolayer, with height 0.9 ± 0.2 nm.

AFM measurements were performed in the semicontact mode to directly measure the height of the monolayer structures. The micrographs and height profiles are shown in Figure 1. The height profiles measured for the pristine and doped samples were 0.8 ± 0.2 and 0.9 ± 0.2 nm, which are within the expected values found in ref (40).

**Figure 2:**
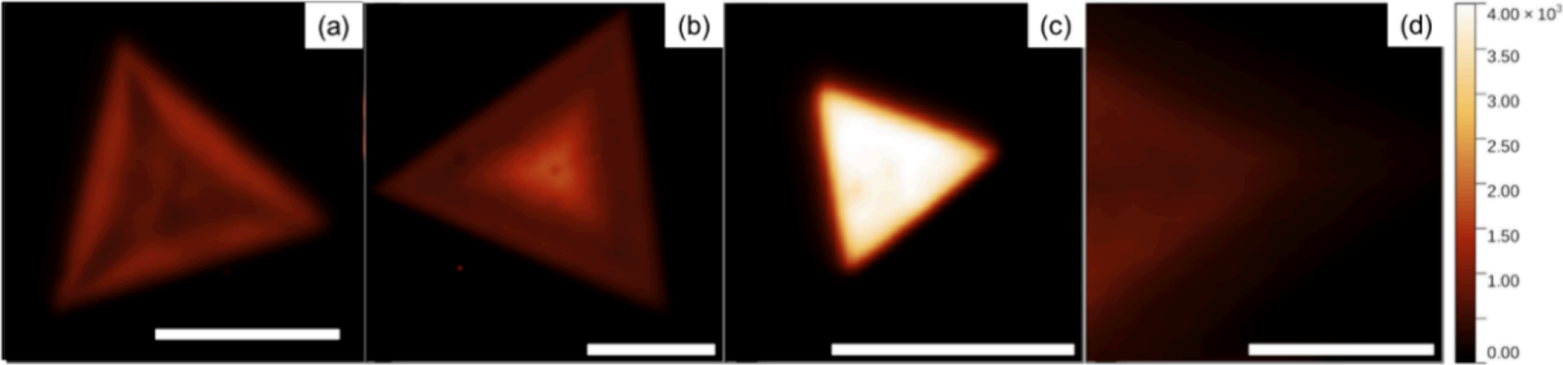
(a–d) PL maps of pristine, 100:1, 2:1, and 1:1 WO_3_:Ga_2_O_3_ mass ratios, respectively. One may observe the intensity gain and uniformity of the 2:1 Ga-doped samples. The scale bar in all of the figures is 10 μm.

**Figure 3:**
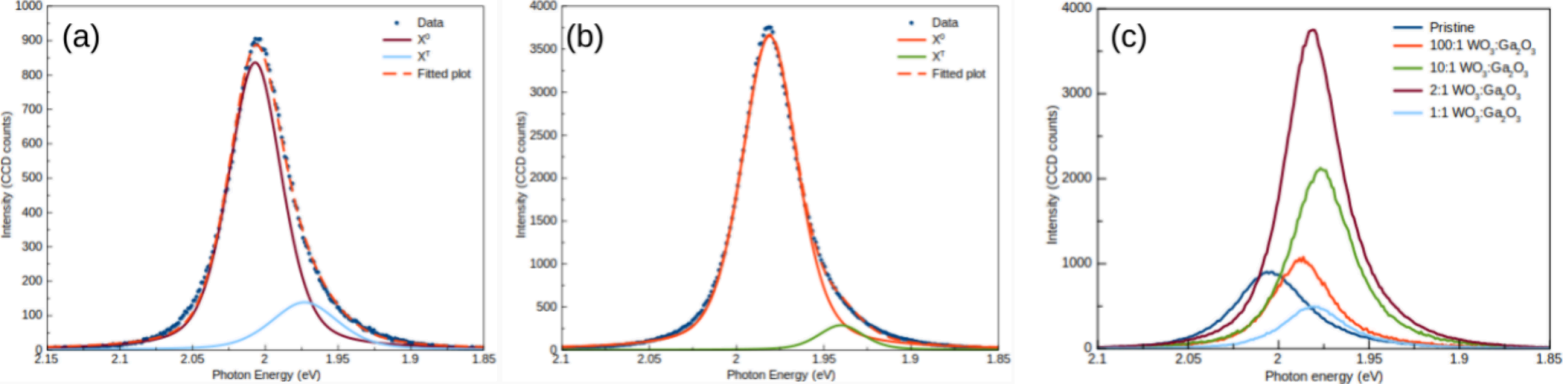
(a, b) PL peak deconvolution showing the *X*^0^ and *X*^T^ contributions for the pristine and *m*(WO_3_):*m*(Ga_2_O_3_) = 2:1 samples, respectively. The smaller contribution of *X*^T^ to the line shape of the emission suggests that the adsorption of gallium enhances the direct recombination process, which directly affects the overall intensity of the PL peak. Panel (c) shows the average PL obtained from samples grown using different mass ratios of WO_3_ and Ga_2_O_3_. There is a displacement of the PL peak due to the adsorption of Ga. The redshift PL observed for the *m*(WO_3_):*m*(Ga_2_O_3_) = 2:1 ratio sample is 26 meV.

Figure 2a–d shows the PL maps of pristine and Ga-doped monolayered samples grown with different W and Ga oxide precursor ratios. The presence of Ga in the growth process did not change the growth dynamics, and the monolayered morphology yielded predominantly triangular structures, as shown in Figure S2. It is also shown, in Figure S4, that the overall peak shift was observed for different monolayer structures, correlating with the data in Table 1. The triangular monolayers doped with Ga showed a more uniform luminescence behavior compared to the pristine ones. The strongest luminescence signal was observed in structures synthesized using a 2:1 *m*(WO_3_):*m*(Ga_2_O_3_) mass ratio. The PL intensity was uniform and, on average, 3.6 times more intense for these doped samples compared with the pristine ones, as is clear from Figures 2c and 3c, as well as from Figure S5 in the Supporting Information. The average intensities were obtained by averaging the mean intensity obtained from the PL maps of several monolayer crystals. It is important to note that the results are an adequate average of several points from different triangles instead of an isolated peak intensity value inside the monolayer triangle. The more intense emission from the edges of pristine samples in Figure 2a is well discussed in the literature.^41^ This phenomenon is due to the high density of S vacancies in the basal plane of WS_2_ monolayers with respect to the edge, where excitonic recombination is more probable.

**Table 1:** PL Peak Parameters as a Function of the WO_3_:Ga_2_O_3_ Mass Ratio

	pristine	100:1	10:1	2:1	1:1
*X*^0^ pos (eV)	2.01	1.988	1.977	1.981	1.981
*X*^T^ pos (eV)	1.970	1.944	1.939	1.940	1.940
*X*^0^/*X*^T^ ratio	6.1	14.6	13.9	12.7	15.1
line shape shift (meV)		19	30	26	26

In Figure 3a,b, the PL peaks are representative of the averaged emission of the structures for the pristine sample and the sample prepared with a mass ratio of WO_3_:Ga_2_O_3_ = 2:1, respectively. We can correlate the average intensity with the precursor powder mixtures, as shown in Figure S5. Figure 3c shows the PL spectra of samples prepared with different mass ratios as well as the PL spectrum obtained from a pristine sample. As is clear from this figure, the presence of Ga also affected the emission energy of the doped samples. It was possible to see a shift of the emission peak up to ∼26 meV upon an increase of Ga in the precursor mixture (Table 1). It is well-known that CVD-grown TMDs are naturally n-doped due to the presence of S vacancies. The observed shift shows that the presence of Ga in the structure dopes the material by changing its emission due to the creation of defect states in the band gap. This is compatible with the process of substitutional doping and may also be responsible for increasing the X^0^ exciton line contribution and intensity,^42^ as well as increasing the *X*^0^/*X*^T^ ratio as shown in Table 1. These changes suggested that Ga atoms are passivating sulfur vacancies, as the density of S vacancies contributes to PL processes via the trion emission. That is, the trion contribution to the line shape is lower for the doped samples, as shown in Figure 3. At low Ga concentrations, sulfur passivation does not immediately contribute to nonradiative processes; however, for highly doped samples, the incorporation of Ga introduces new defect states, increasing the concentration of sites where nonradiative recombination processes may take place. This is a recombination pair that enhances the Auger recombination process,^43^ which is not related to trions or neutral excitons. This effect may be due to the strong alloying of the monolayer structures or an effect of strain due to augmented lattice deformation in the presence of Ga impurities.

A recent report revealed an enhancement of PL intensities seen from the fluorescent spectra of Er-doped WS_2_ monolayers compared with those of the pristine samples. In this case, Er atoms are substitutional in W sites.^44^ In the case of Nb p-doped WS_2_ grown by CVD, Nb atoms also substituted the W atom in the WS_2_ lattice but quenched the PL emission.^45^

**Figure 4:**
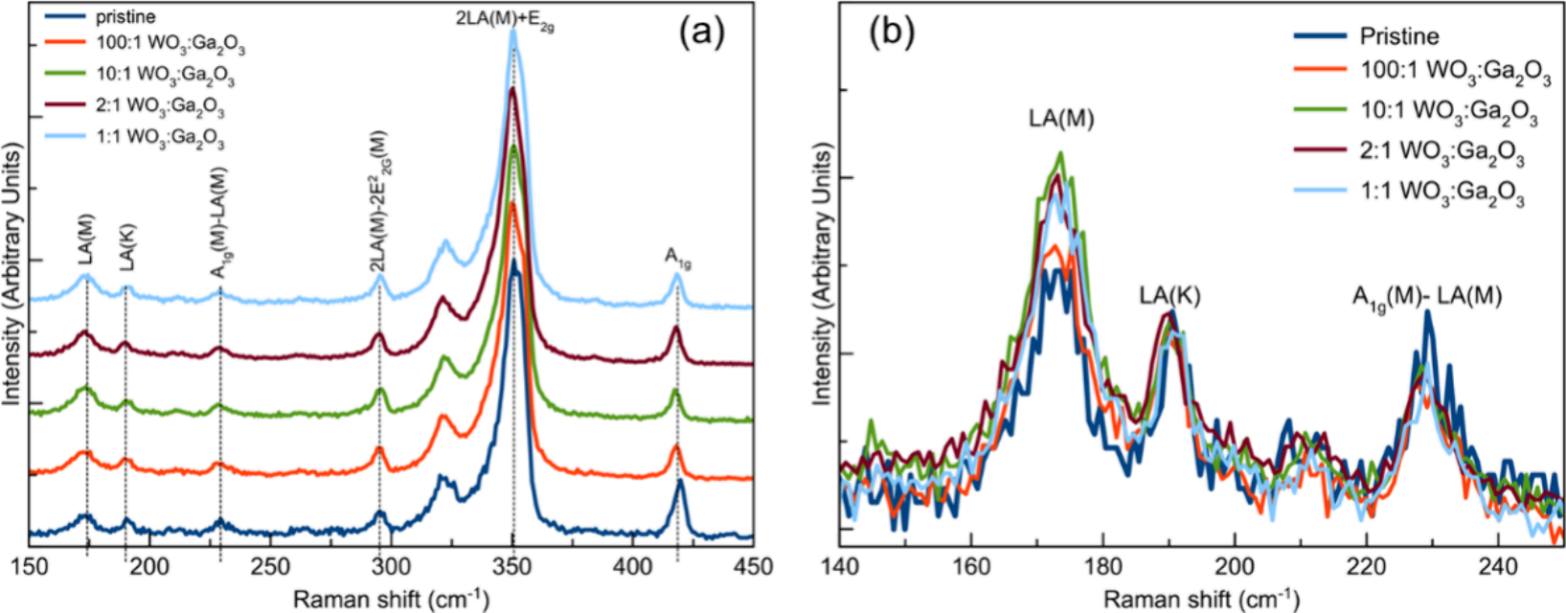
(a) Raman spectra of pristine and Ga-doped samples synthesized with different mass ratios. It is possible to observe a shift in the A_1g_ peak of around 0.7 cm^–1^ for all doped samples. (b) Lower-frequency spectral region. The LA(M) vibrational mode (176 cm^–1^) increases due to increasing disorder effects in the samples, a behavior not followed by the LA(K) (190 cm^–1^) and A_1g_(M)-LA(M) (224 cm^–1^) vibrational modes. The excitation wavelength was 532 nm.

Figure 4a shows the full-range Raman spectra of pristine and Ga-doped WS_2_ monolayers. Raman spectra of the WS_2_ crystal show two main peak contributions: the in-plane 2LA(M)+E_2g_ mode at ∼355 cm^–1^ and the out-of-plane A_1g_ mode at ∼419 cm^–1^. Indeed, the second-order 2LA(M) convolved with the E_2g_ mode is the most intense feature of the spectra. It is expected that the A_1g_ mode is sensitive to doping effects.^46^ A small shift toward lower frequencies and softening of the A_1g_ mode were seen in the Ga-doped samples, as shown in Figure 4a; also, off-resonance measurements did not show a peak shift in the E_2g_ mode, indicating the samples were not affected by strain effects due to Ga incorporation (Figure S6). This minor change may be mostly due to out-of-plane vibrations affected by the presence of Ga impurities in the basal plane of the crystal interacting with the top S layer. The A_1g_ redshift is not an effect of induced heat or damage during the measurements because the reproducibility of the spectra was tested at different laser powers without any change, or any effect of strain, which would strongly affect the line shape in the 2LA(M) spectral region due to the splitting of the E_2g_ mode in the two contributions. Hence, the Raman spectra evidence the presence of Ga in the lattice as well as indicate a p-type doping effect,^47,48^ which is also confirmed by the energy shift in the position of the valence band maximum (VBM), shown in Figure S10.

However, at an excitation wavelength of 532 nm, one can observe more features due to the resonance Raman effect. This effect is due to the excitation laser energy resonance with the *X*^b^ exciton of WS_2_, which shows an absorption peak at around 520 nm (∼2.4 eV).^49^ Specifically, disorder-related modes in the lower-frequency part of the spectrum were seen.^50^ They may be used to infer the influence of the presence of impurities in the lattice, as is the case of the heteroatoms, or as the change of the density of defects in the material compared to the pristine samples.^48^ It is shown in Figure 4b that the intensity of the LA(M) vibrational mode at 176 cm^–1^ increases upon the increase of the Ga_2_O_3_ concentration in the precursor mixture.

**Figure 5:**
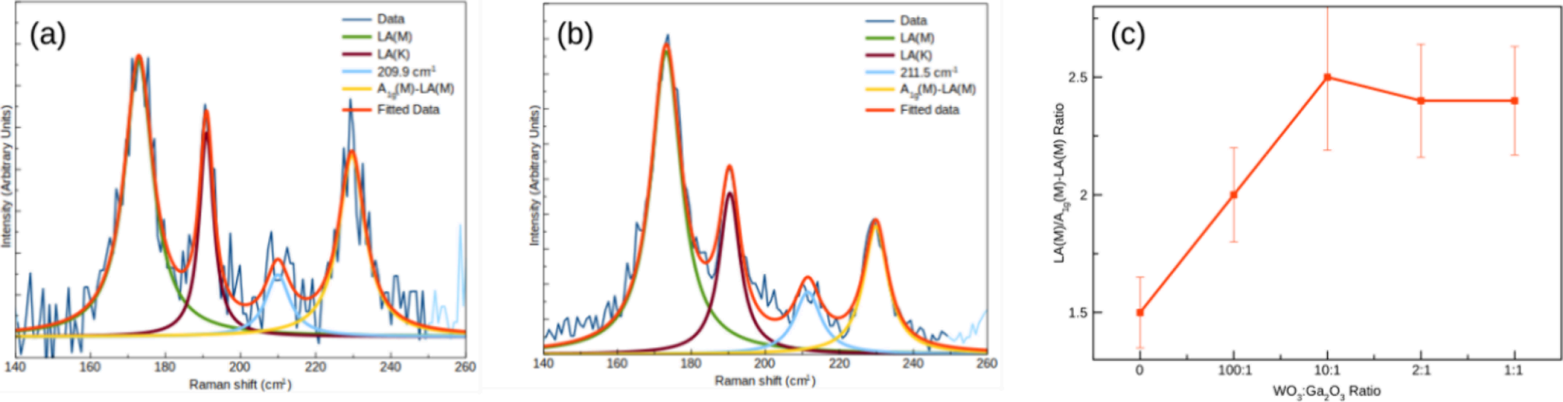
(a) Lower-frequency Raman spectrum from the pristine WS_2_ monolayer sample. (b) Same spectral region, in this case for the 2:1 Ga-doped sample (the WO_3_:Ga_2_O_3_ mass ratio is 2:1). (c) Evolution of [LA(M)/(A_1g_(M)-LA(M)] as a function of the WO_3_:Ga_2_O_3_ mass ratio. The excitation wavelength was 532 nm.

In Figure 5a,b, we present the low-frequency Raman spectra obtained from a pristine sample and a monolayer prepared with an *m*(WO_3_):*m*(Ga_2_O_3_) mass ratio of 2:1, respectively. Further, the LA(M)/[A_1g_(M)-LA(M)] intensity ratio increases from 1.5 to 2.4, as shown in Figure 5c. The error in this ratio is around 10%. This ratio is of the most importance to qualitatively verify the increase in the density of defects in the treated samples as the A_1g_(M)-LA(M) combined mode intensity remains nearly constant across all samples. Considering that heteroatoms induce disorder effects, this result also suggests the presence of Ga atoms in the crystal lattice.

As seen in Table 2, where we list the position of the main features of the low-frequency Raman spectrum, an overall spectral shift, if any, was not observed, and any conclusion based on a position change of the peaks cannot be made, despite the increase of the density of defects as a result of the presence of Ga atoms in the lattice structure, as suggested in Figure 5c.

**Table 2:** Position Parameters and Peak Ratio of the Pristine and Ga-Doped Samples

	pristine	100:1	10:1	2:1	1:1
LA(M) pos (cm^–1^)	172.8	173.44	173.9	173.2	173.2
LA(K) pos (cm^–1^)	190.9	191.33	191.7	190.4	191.1
A_1g_(M)-LA(M) pos (cm^–1^)	229.7	230.2	230.1	229.9	229.4
LA(M):[A_1g_(M)-LA(M)] ratio	1.5	2.0	2.6	2.4	2.4

**Figure 6:**
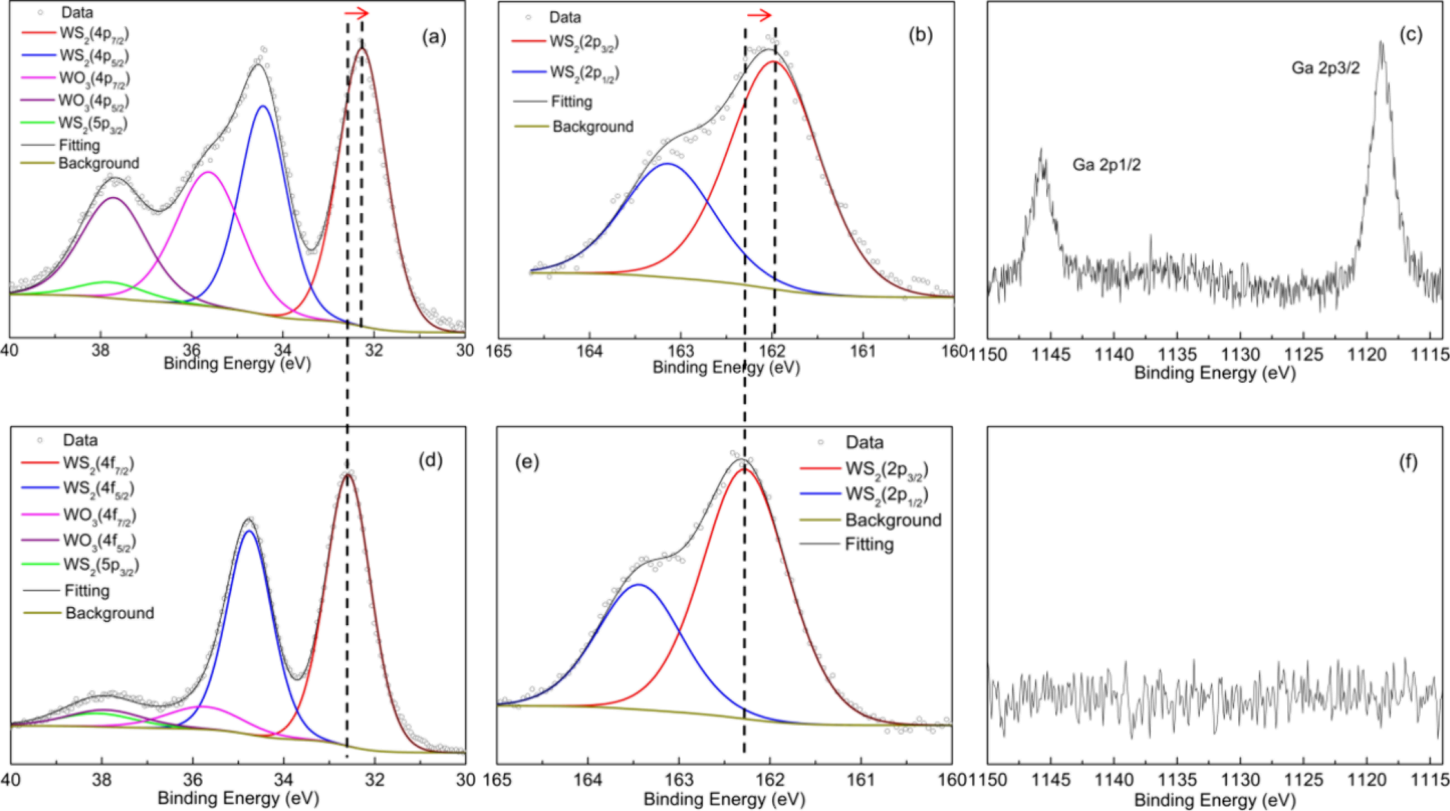
(a–c) XPS spectra of W4f, S2p, and Ga2p of Ga-doped WS_2_ samples prepared with a 2:1 WO_3_:Ga_2_O_3_ mass ratio; (d–f) spectra of W4f, S2p, and Ga2p of pristine WS_2_ samples. There is a clear shift toward lower binding energies for the Ga-doped WS_2_ sample, as is shown by the dashed line and arrow in the figure.

To ascertain the presence of Ga atoms in WS_2_ monolayers, XPS was employed for analysis. The XPS spectra of Ga-doped WS_2_ and pristine WS_2_ are presented in Figure 6. Specifically, Figure 6a–c illustrates the W4f, S2p, and Ga2p regions of the Ga-doped sample prepared with a 2:1 WO_3_:Ga_2_O_3_ mass ratio as the precursor. In contrast, Figure 6d,e displays the W4f, S2p, and Ga2p regions of pristine WS_2_ samples. Analysis of Figure 6a,b reveals that, in the Ga-doped sample, the W4f and S2p peaks are shifted to lower binding energies by 0.41 and 0.40 eV, respectively, compared to the pristine sample (Figure 6d,e). The redshift of W4f and S2p peaks in the Ga-doped WS_2_ samples indicates a shift of the Fermi level toward the valence band due to the presence of Ga atoms. From the analysis of the XPS spectra, we were able to determine the ratio between S and W atoms (S/W) as being equal to 2.10 ± 0.15 for pristine samples and 1.90 ± 0.15 for samples synthesized using as precursor a mixture of oxides with a mass ratio of 2:1. To obtain this (S/W) ratio, only W atoms bonded to S atoms were considered, indicating that both pristine and Ga-WS_2_ samples were nearly stoichiometric. It is also important to note that there is no shift in the position of the WO_3_ peaks, showing that the oxide was not incorporated in the samples. The binding energy peaks of Ga2p_1/2_ and Ga2p_3/2_ are distinctly seen in the Ga-doped WS_2_, while no meaningful signals are present in the same region for pristine WS_2_, as depicted in Figure 6c,f. This observation serves as direct evidence of the presence of Ga in the Ga-WS_2_ samples.

**Figure 7:**
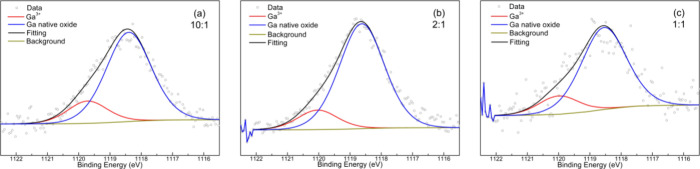
Deconvoluted Ga2p_3/2_ XPS spectra of doped samples prepared with different WO_3_:Ga_2_O_3_ mass ratios of 10:1 (a), 2:1 (b), and 1:1 (c).

A detailed analysis of the Ga peaks is presented in Figure 7. The content of Ga in the sample prepared with a 100:1 *m*(WO_3_):*m*(Ga_2_O_3_) ratio was below the detection limit of our XPS system. For all mass ratios analyzed, the XPS spectra were deconvolved into two peaks: one at 1120.1 eV (fwhm = 1.75 eV) and the second at 1118.5 eV (fwhm = 1.70 eV), the latter being the dominant one. When the spectra are interpreted, we can rule out the presence of both metallic Ga and Ga_2_O_3_. This is because the position of the bands corresponding to them would be 1116.7 eV (fwhm = 1.04 eV)^51,52^ and 1117.7 eV (fwhm = 1.53 eV),^52,53^ respectively. Based on results reported in the literature, we attributed the band at 1118.8 eV to the presence of Ga native oxide and the band at 1120.2 eV to Ga^3+^.^51,54^ These results substantiate the adsorption of Ga into the WS_2_ structures, thereby influencing the electronic properties.

In Table 3, we present the contents of W, S, and Ga^3+^ as well as the ratio W/S. The amount of Ga^3+^ increases with the increase of Ga in the mixture. In effect, the total amount of Ga increases from 2% up to 10% (not shown in Table 3), and for the case of samples prepared with equal masses of the WO_3_ and Ga_2_O_3_ ratio, this can explain the observed reduction of PL intensity. In fact, for these oxide masses, the W/S ratio (1.5) is another indication of the loss of quality of the crystal structure and, consequently, the observed reduction in PL.

**Table 3:** Chemical Composition of the Samples, Considering the Contribution of W and S from Figure 6 and the Ga^3+^ from Figure 7

	pristine	10:1	2:1	1:1
Ga^3+^ (at %)		0.5	1.3	1.5
W (at %)	32.1	32.9	34	39.1
S (at %)	67.9	66.6	64.7	59.5
S:W ratio	2.1	2	1.9	1.5

The VBM of both pristine and 2:1 Ga-doped WS_2_ samples is presented in Figure S10, determined using the linear extrapolation method. A shift toward lower binding energies indicates that the Fermi level is moving closer to the VBM, suggesting p-type doping as a result of Ga incorporation. This observation is consistent with the Raman spectroscopy results.

To confirm the scenario suggested by the XPS and Raman results, DFT calculations were performed. They were conducted by the Vienna ab initio simulation package (VASP).^37^ Information on the computational details can be found in the Supporting Information. The formation energies (*E*_F_) were obtained following the procedure of Kieczka et al.^39^ as follows:
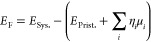
1

Here, *E*_F_ is the defect formation energy, *E*_Sys._ is the energy of the system with the defect, *E*_Prist._ is the energy of the system without the defect, μ_*i*_ is the chemical potential, and *n*_*i*_ is an index that can be +1 or −1 depending on the addition or subtraction of an atom into the lattice, respectively. The systems considered were the Ga atom as a substitutional impurity replacing W (Ga_W_) or S (Ga_S_) and the scenarios where one Ga atom is adsorbed above the W atom (Ad_W_), above the S atom (Ad_S_), or above the hexagonal center (Ad_H_). In the Supporting Information, we describe in detail how the chemical potentials were calculated, and we also present a figure describing the positions of the adsorbed atoms. In the case of adsorbed impurities, the smaller *E*_F_ is 6.10 eV for the Ad_W_ system. For the Ad_H_ and Ad_S_ systems, we obtained *E*_F_ equal to 6.19 and 6.32 eV, respectively. When substitutional impurities are considered, *E*_F_ depends on the W and S chemical potentials. Thus, depending on the experimental conditions, we get different values for *E*_F_. For S-rich conditions, we obtained *E*_F_ equal to 5.52 and 8.65 eV for Ga_W_ and Ga_S_, respectively. On the other hand, for W-rich conditions, we obtained *E*_F_ equal to 8.54 and 7.13 eV for Ga_W_ and Ga_S_, respectively. Simulations thus confirm that Ga is likely to be adsorbed, while it could be substituted depending on the deposition process.

Noteworthy that despite the adsorption cases and the Ga_W_, under S-rich conditions, being the formed configurations, the localized states formed in the latter suggest that the PL can be red-shifted. Likewise, we would like to point out that our simulations show that the adsorption of Ga will interact with the top S layer, which agrees with the shift and softening of the modes of the Raman spectrum.

**Figure 8:**
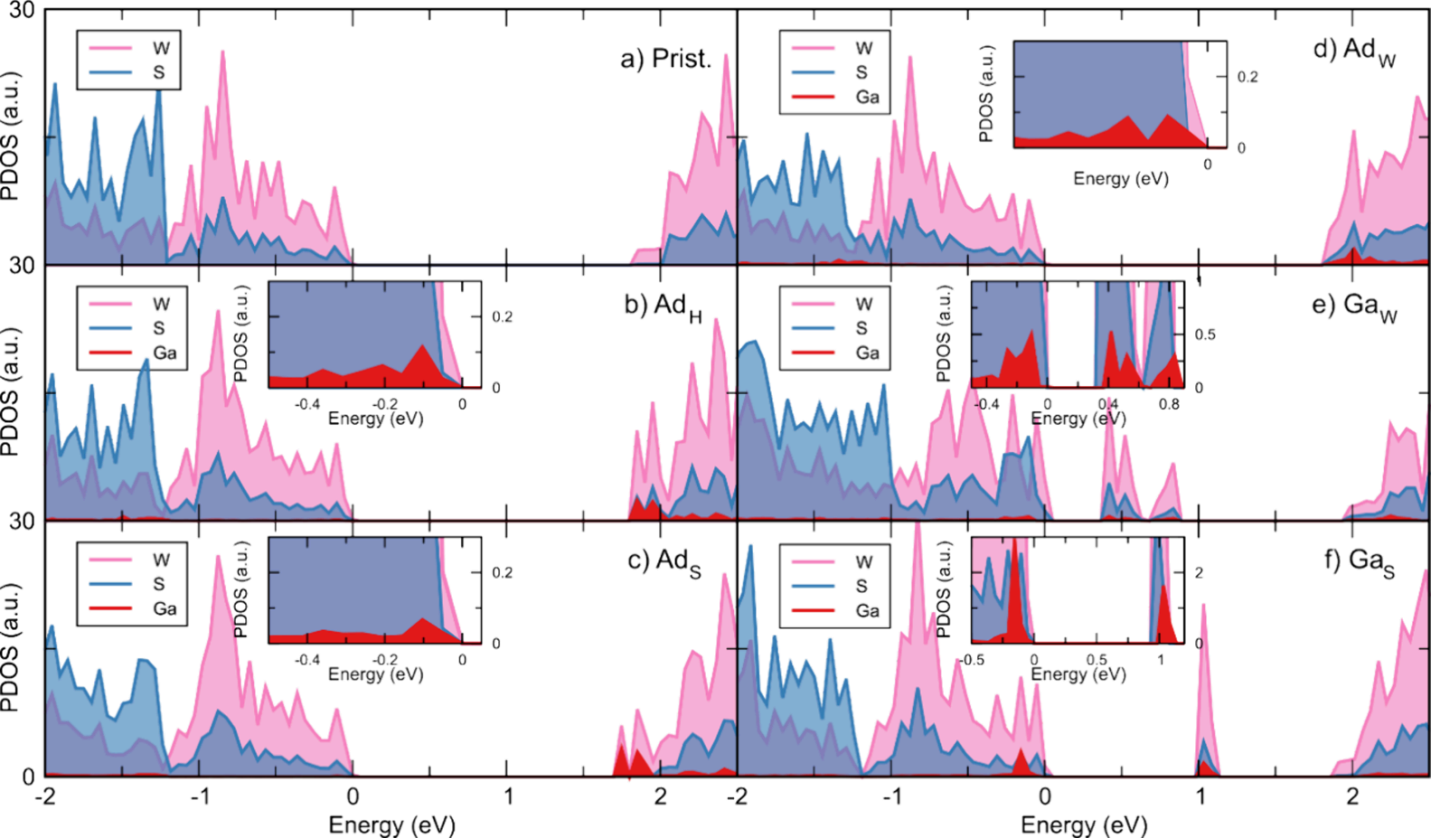
Projected density of states. (a) Pristine WS_2_, (b) Ad_H_, (c) Ad_S_, (d) Ad_W_, (e) Ga_W_, and (f) Ga_S_. Insets show an augmented view of the Ga impurity states near the band edge.

In Figure 8, the projected density of states of the configurations is shown. Here, we notice that the Ga impurities introduce states in the electronic gap for the Ga_S_ and Ga_W_ substitutional cases. However, in other cases, it introduces states near the top of the valence band. This agrees with our experimental results of the Ga introducing a p-type degeneration. To support this idea, we show the atomic net charges calculated in Table 4, from the DFT electronic density, using the Bader approach.^55,56^ For pristine WS_2_, as expected, W atoms lose around 1.2 electrons to the surrounding S atoms. In the case of systems with Ga impurities, we present in the table the average of the Bader net charges for the atoms in the supercell. In these cases, we see that the Ga impurities always lose electrons to the WS_2_ matrix.

**Table 4:** Atomic Net Charges Calculated from the DFT Electronic Density Using the Bader Approach^a^^52,53^

	Bader net atomic charge		
	W (avg.)	S (avg.)	Ga
Ad_H_	+1.234	–0.634	+0.537
Ad_W_	+1.224	–0.631	+0.605
Ad_S_	+1.225	–0.626	+0.424
Ga_W_	+1.251	–0.622	+1.120
Ga_S_	+1.181	–0.622	+0.393
prist. WS_2_	+1.239	–0.619	

a For W and S atoms, the average net charges are presented.

The electron transfer from Ga impurities to WS_2_ can be explained by the differences in electronegativity and ionization potential of W and S with Ga atoms. In Figure S9 of the Supporting Information, the electronegativities and ionization potentials are displayed. Interestingly, in ref (57). Christopoulos et al. calculated the Bader net charges in doped Si, Ge, and Si_1–*x*_Ge*_*x*_*. They show that for typical p-type impurities such as Al, Ga, and In, there is an electron transfer from the impurity to the hosting material.

## 4 Summary and Conclusions

In summary, our work showed that the in situ doping strategy effectively incorporates Ga impurities to WS_2_ monolayers, in which its most significant effect is an enhancement of the luminescence intensity of up to 3.6 times when compared to pristine WS_2_ with similar triangular morphologies, where the main contribution to the PL line shape arises from the neutral exciton recombination, which is evidence of Ga atoms patching sulfur vacancies. Also, Raman spectroscopy revealed that the presence of Ga affects the crystals’ vibrational properties, where disorder-related effects are directly affected and where the LA(M)/[A_1g_(M)-LA(M)] peak intensity ratio increased due to the presence of Ga impurities in the lattice. In particular, the small redshift observed in the A_1g_ mode indicates that Ga species are p-doping the WS_2_ monolayers, which is compatible with the literature results seen elsewhere. Finally, XPS corroborates this by showing a clear energy redshift in the W_4f_ and S_2p_ regions. Moreover, the observed evolution in the Ga_2p_ region serves as compelling evidence, indicating a correlation of the amount of Ga incorporated into the lattice with respect to the precursor proportion ratio. Both XPS results and DFT calculations agree that Ga is mostly adsorbed on the WS_2_ surfaces, while a small percentage of Ga was incorporated into the crystal lattice at the structure on the W sites.

## Supporting Information Available

CVD growth method and conditions; optical microscopy of grown samples; PL spectra of precursors; supplementary PL data; off-resonance Raman spectra; computational details of DFT calculations; and supplementary XPS data (PDF)
