# Supplementary material for: Significant Luminescence Enhancement of Ga-Doped WS2 Monolayers Grown by CVD
Source: ACS Omega. 2025 Apr 9;10(15):15663–72. doi: 10.1021/acsomega.5c01066 (PMC12019728; doi:10.1021/acsomega.5c01066)
Supplement: Supplementary file 1 — ao5c01066_si_001.pdf [file ao5c01066_si_001.pdf]

Supplementary Material for

"Significant luminescence enhancement of Ga-doped WS_2 monolayers grown by CVD"

by

Shuai Zhang[1], Andre N Barbosa[1*], Munique Eva Paiva de Araujo Monteiro de Barros[2], Alexandre Mello[2], Kevin Lizárraga[3,4], Pedro Paulo de Mello Venezuela[3], Fernando Lázaro Freire Jr[1]

[1] Department of Physics, Pontifícia Universidade Católica do Rio de Janeiro, 22451-900, Rio de Janeiro, RJ, Brazil.

[2] Laboratory of Surfaces and Nanostructures, Brazilian Center for Physics Research, 22290-180, Rio de Janeiro, RJ, Brazil.

[3] Institute of Physics, Fluminense Federal University, 24210-346, Niteroi, RJ, Brazil.

[4] Departamento de Ciencias, Sección Física, Pontificia Universidad Católica del Perú, Av. Universitaria 1801, Lima 32, Peru.

*Corresponding author email: andrenbarbosa@vdg.fis.puc-rio.br

Apparatus and conditions for synthesis

Fig. S1 illustrates the schematic representation and photograph of the synthesis system, including the temperature profile during the heating process. Notably, the temperature of the resistance section is verified using a thermometer (Model: Minipa MT401A), with its probe positioned inside the quartz tube within the resistance section.

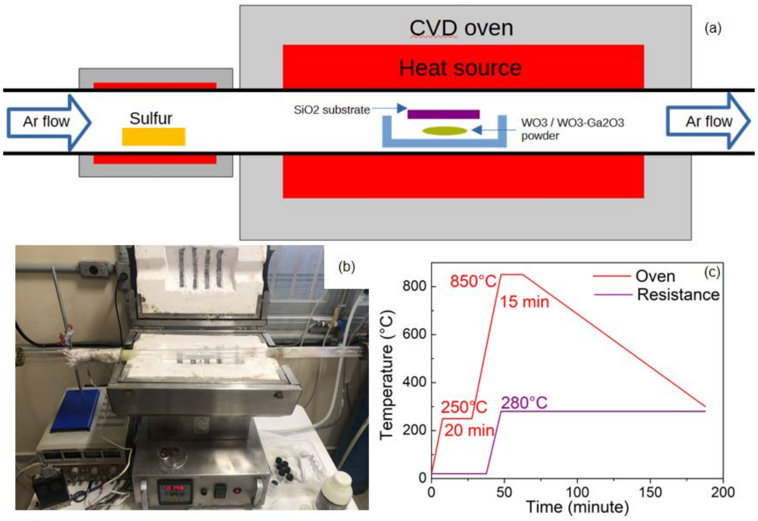

Fig. S1. The scheme of synthesis system of pristine and Ga-doped WS_2 is demonstrated as (a), and the real equipment photo is shown as (b). (c) shows the temperature profile of the synthesis process both in oven (red line) and resistance covered part (purple line).

The melting point of not only the Ga precursor (1725°C), but also the W precursor (1473°C) is far beyond the synthesis temperature used in this work, if we only consider the temperature to break down the transition metal oxides. However, one must consider the sulfurization process that occurs for both oxides at lower temperatures. In fact, it is present in typical WS_2 growth in the case of WO_3 precursor, as well as for Ga_2O_3 nanowires at even lower temperatures [1,2].

Optical images of WS_2

Optical images of pristine and Ga-doped WS_2 sample are shown in Fig. S2. The size of WS_2 ranges from 2 μm to more than 100 μm, but most samples are 5 to 50 μm in size.

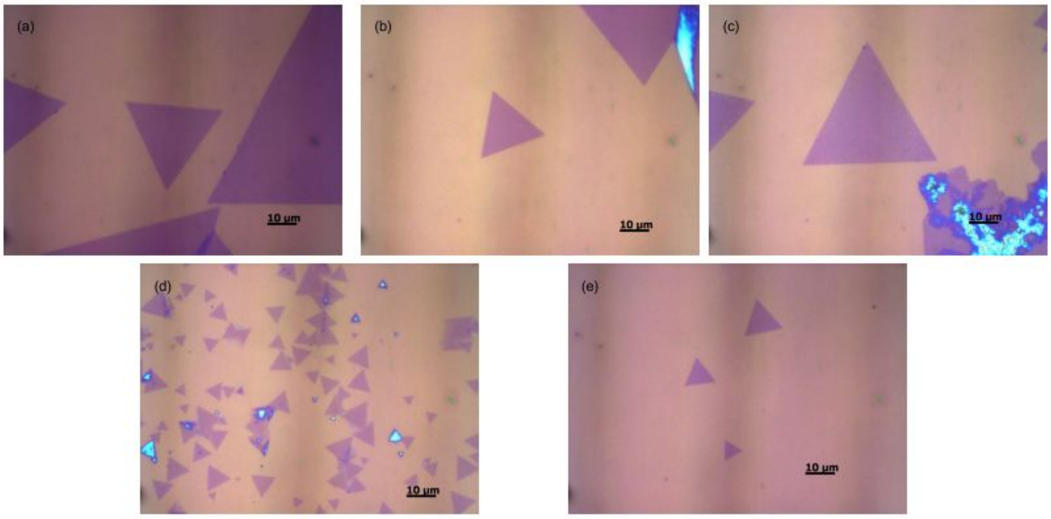

Fig. S2. Optical images of pristine (a) and Ga-doped WS_2 samples with different WO3:Ga2O3 mass ratio, 1:1 (b), 2:1 (c), 10:1 (d) and 100:1 (e), respectively.

Photoluminescence of WO_3 powder

To eliminate the interference from reactants (WO_3 and Ga_2O_3) to the product (WS_2), the photoluminescence of WO_3 and Ga_2O_3 powder is also assessed. Analysis of their photoluminescence spectra reveals an absence of any meaningful peak in the vicinity of the 1.8-2.1 eV range, where the primary peak in the photoluminescence of WS_2 is situated.

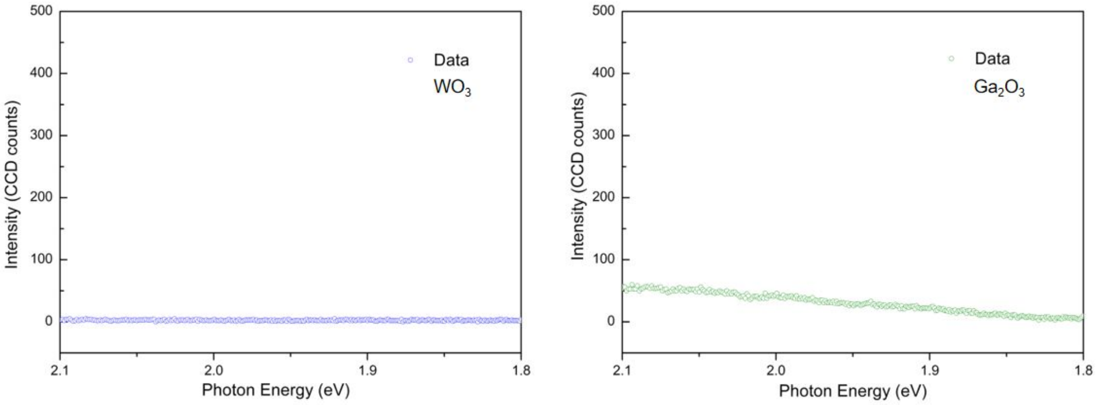

Fig. S3. PL spectra of WO_3 and Ga_2O_3. No obvious PL peaks are located at around 1.8-2.1 eV range.

A statistical analysis was performed to investigate the average peak position. It is possible to see in Fig. S4 that the peak wavelength variation remains uniform, unlike the PL intensity uniformity, as shown in Fig.2. Table 1 highlight the mean wavelength and variation and mean FWHM of the PL lineshape. The analysis show that the peak wavelength variation is significantly smaller than the FWHM of the mean wavelength linewidth, which indicates the uniformity of the peak emission wavelength.

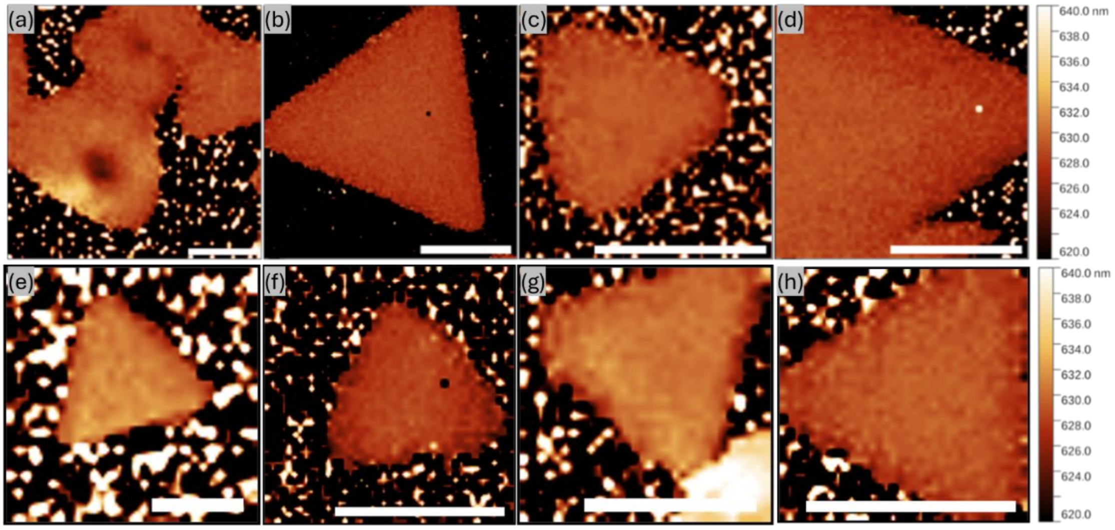

Fig. S4. Peak wavelength maps of pristine and doped samples. (a) and (e) are pristineWS_2 monolayer structures, (b) and (f) are 100:1 Ga-WS_2, (c) and (g) are 2:1 Ga WS_2, and (d) and (h) are 1:1 Ga-WS_2 samples. The scale bar in wall the figures is 10 μm.

Table S1. PL line shape peak position and parameters:

WO_3:Ga_2O_3 ratio	Average position (nm)	Average FWHM (nm)	Position variation (nm)
Pristine	619.9	18.2	-
100:1	623.7	12.1	1.3
2:1	625.9	14.1	1.3
1:1	625.9	11.2	1.3

Average PL intensity of WS_2 monolayers

We have performed PL maps in several different monolayer structures (including monolayer triangles and monolayer films), grown in different substrates (at least three to confirm repeatability).

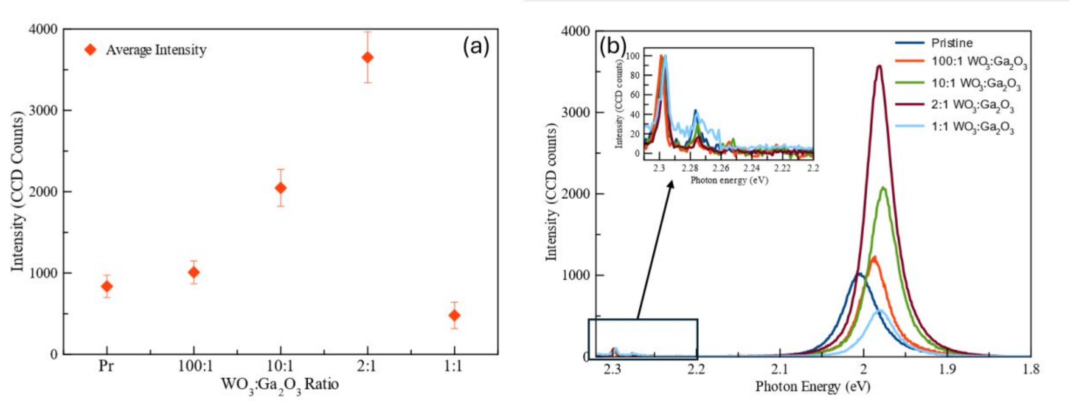

Fig. S5: (a) Average PL intensity of the measured monolayers with different m(WO_3):m(Ga_2O_3) ratios. (b) PL spectrum showing the 2LA(M)+E_{2g} Raman peak with same intensity for all measurements.

Off-Resonance Raman spectroscopy

Raman spectroscopy was performed using an laser with excitation wavelength of 473 nm, where the energy (2.6 eV) lies far from an electronic transition, significantly quenching second order and other modes only present in Resonance Raman conditions. Hence, only the main out-of-plane (A_{1g}), and in-plane (E_{2g}) modes are prominent in the spectra. In this case, it is possible to observe that the is not a observable shift due to strain effects in the in-plane (E_{2g}) mode, indicating that Ga incorporation is not strongly affecting the mechanical properties of the material. In fact, a small red shift was observed in the A1g mode, which indicates a doping effect due to Ga incorporation.

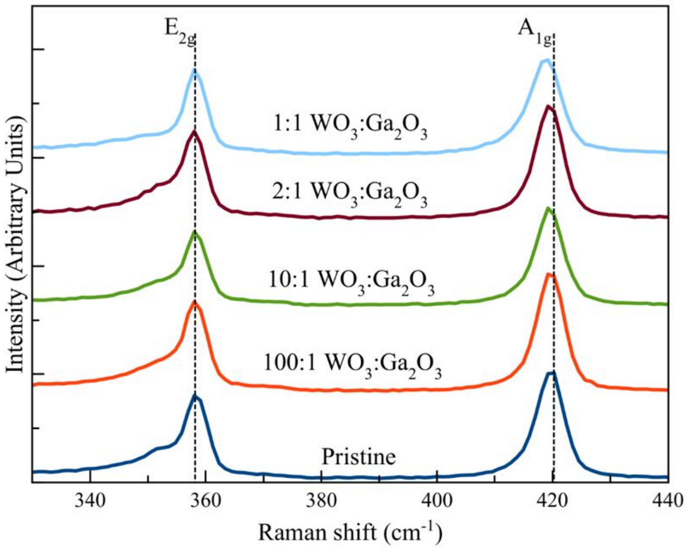

Fig. S6. Raman spectrum of pristine and Ga-WS_2 grown with different WO_3:Ga_2O_3 ratios. Within the spectrometer margin of error, only a shift in the A_{1g} mode is observed, which indicates a doping effect. The spectra were acquired using a 473 nm laser.

Table S2. Peak position and FWHM of the main Raman spectra modes (473 nm laser)

Sample	A_{1g}		E_{2g}	
	Pos (cm^{-1})	FWHM (cm^{-1})	Pos (cm^{-1})	FWHM (cm^{-1})
Pristine	419.3	4.3	358.1	3.8
100:1	418.6	4.2	358.0	4.0
10:1	418.5	4.3	358.3	3.8
2:1	418.3	4.2	358.2	4.2
1:1	418.3	4.2	358.3	3.9

Computational details of the DFT calculations

We have performed density functional theory (DFT) calculations using the Vienna Ab Simulation Package (VASP) code [3]. For the exchange-correlation functional we used the Perdew–Burke–Ernzerhof (PBE) generalized gradient approximation including Tkatchenko-Scheffler dispersion corrections (IVDW=20) [4] and magnetic corrections (ISPIN=2, LNONCOLLINEAR=.FALSE.). A cutoff energy of 500 eV and a 3x3x1 grid of k-points has been used for relaxing all structures. The energy and forces threshold for the relaxation were set to 10^{-6}eV and $5x10^{-3}$eV/$\dot{A}$.

Our pristine structure contains 48 atoms as can be seen from Fig. S7 (a) and (b). This is a result of the 4x4x1 replication of the WS_2 unit cell. Consequently, the size of the cell is 12.72$\dot{A}$ in length for the xy sides, while in the zth direction it contains 23.2$\dot{A}$ of vacuum for neglecting layers interaction. Illustrations of the systems containing the Ga impurity can be seen in Fig. S4 (c)-(f). Here, the adsorption sites correspond to Fig. S4 c (Ad_H), d (Ad_S) and e (Ad_W), while the substitutional cases are presented in Fig. S4 f (Ga_W) an h (Ga_S). In addition, we have plotted (see Fig. S4 (g) and (i)) the bond length of substitutional cases for contrasting with the pristine case. The pristine bond length of 2.42$\dot{A}$ is increased to 2.47$\dot{A}$ for the Ga_W, while a quenching is observed for the W-S bond on the Ga_S structure.

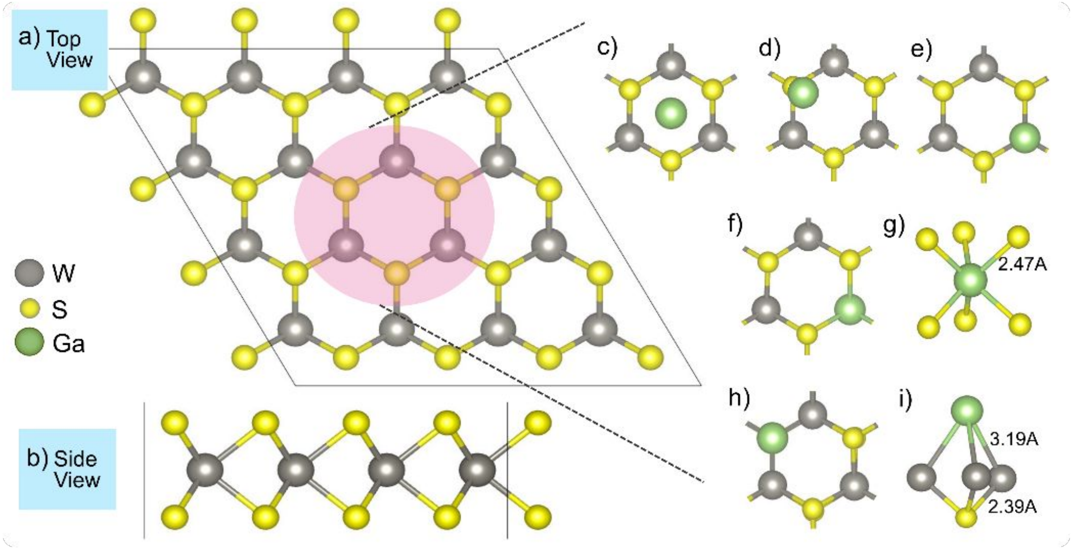

Fig. S7. WS_2 Structures for the different configurations. The Pristine WS_2 structure is presented in (a) and (b) from the top and side views, respectively. Ga doped configurations are ordered in the following. Ad_H (c), Ad_S (d), Ad_W (e) Ga_W (f) and Ga_S (h). The side view and bond-length of Ga_W and Ga_S are shown in (g) and (i), respectively.

For the convergence of the defect formation energy, we have used supercells of 4x4x1 and 5x5x1 for which we have observed negligible changes in the order of meV.

Calculations of the formation energies were performed following the approach of Kieczka et al. [5], in which the defect formation energy is calculated according to:

$$E_F = E_{Def.Sys} - \left(E_{Prist} + \sum_i n_i \mu_i \right)$$

Here E_F is the defect formation energy, $E_{Def.Sys}$ is the energy of the system with defect, E_{Prist} is the energy of the system without defect, μ_i is the chemical potential, and n_i is an index that can be +1 or -1 depending on the addition or subtraction of an atom from the lattice, respectively.

Chemical potentials of Ga, S and W were calculated from the following. In the case of Ga, we have used the energy of the Ga_2O_3 and subtract the energy of an isolated oxygen molecule, i.e.,

$$\mu_{Ga} = \frac{1}{2} E_{Ga_2O_3} - \frac{3}{4} E_{O_2}$$

For the cases of W and S, the chemical potential changes according to the conditions of the experiment. For instance, in the case of S rich environment in which W atmosphere becomes poor, we have:

$$\mu_S^{rich} = \frac{1}{2}E_{S_2}$$

$$\mu_W^{poor} = E_{WS_2} - E_{S_2}$$

Here, energies of disulfide were calculated on isolated environment. On the opposite, the case where the environment is favorable for W and poor for S, the chemical potential becomes:

$$\mu_W^{rich} = E_{Bulk-W}$$

$$\mu_S^{poor} = \frac{1}{2}(E_{WS_2} - E_{Bulk-W})$$

The chemical potentials obtained in our calculations are comparable to the ones obtained in Kieczka et al. [5]. These are -3.58 meV and -17.13 meV for the μ_S^{rich} and μ_W^{poor}, respectively. While values of -5.09 meV and -14.10 meV were obtained for μ_S^{poor} and μ_W^{rich}, respectively. And, for the Ga atom, we have obtained $\mu_{Ga} = -7.64$ meV.

Thus, the formation energy of the adsorbed cases was 6.187 meV, 6.101 meV and 6.319 meV for Ad_H, Ad_W and Ad_S, respectively. On the other hand, for the substitutional case of Ga_W, we have 8.544 meV and 5.517 for W-rich and S-rich conditions, respectively. Meanwhile, for the substitutional case of Ga_S, we have 7.134 meV and 8.648 for W-rich and S-rich conditions, respectively.

For testing the reliability of our DFT calculations, we have also calculated the formation energies of the pristine system (WS_2) with W vacancy (V_W) and S vacancy (V_S), for comparison purposes only. Fig. S8 shows the comparison of our results in comparison to the one performed by Kieczka et al. [5].

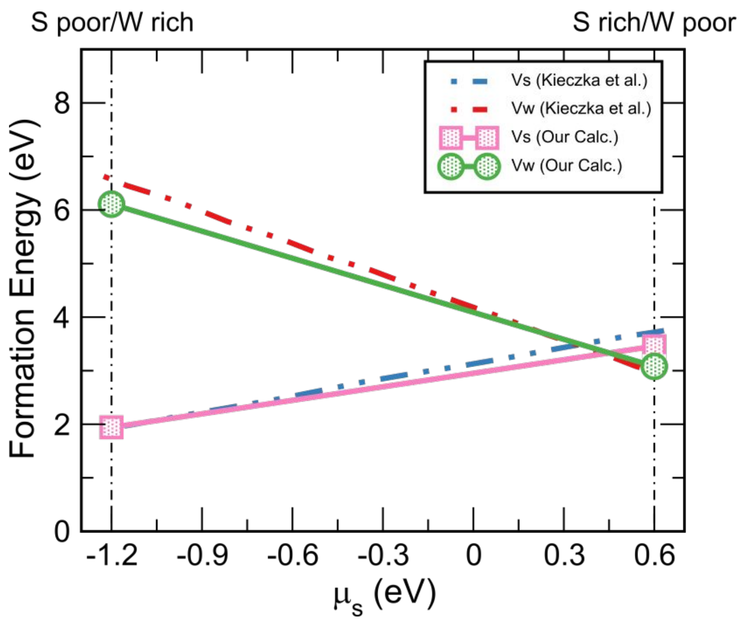

Fig. S8. Shows the formation energies of the vacancies of W and S in the WS_2 matrix under rich and poor conditions. These results are compared with the ones obtained by Kieczka et al. [5].

Electronegativity and Ionization energy of W, S and Ga

To understand the p-type nature of the Ga in the WS_2 matrix, the electronegativities and ionization energies of W, S and Ga are reported in Fig. S7 (a) and (b), respectively. In Fig. S9 (a), the Ga atom has a larger difference in electronegativity with respect to S than to W (see the yellow bar), so, the S atom will be more likely to gain electrons in all the configurations. The same happens for the differences in ionization potential (See Fig. S9 (b)). So, we end up with trends of negative differences of Ga-W and Ga-S in electronegativity/ionization potential and positive Bader charges (See red color bars) for all the configurations.

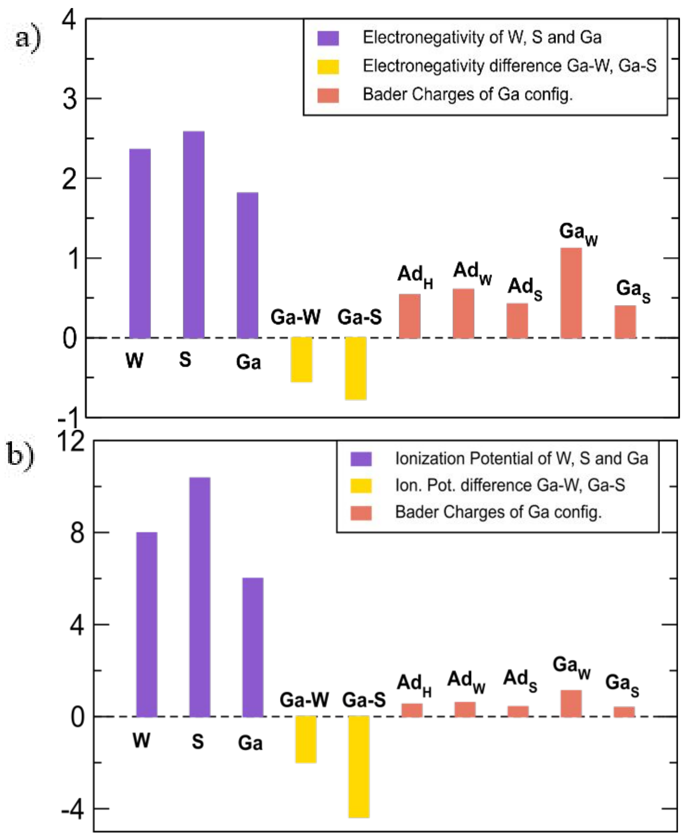

Fig. S9. Show the electronegativities (a) and ionization potential (b) of W, S and Ga. In both figures, the relative differences of W-Ga and S-Ga is shown in yellow color bars. And, the Bader charges of the configurations in which Ga is being adsorbed or is a substitutional impurity are presented as red color bars.

Valence band maximum (VBM)

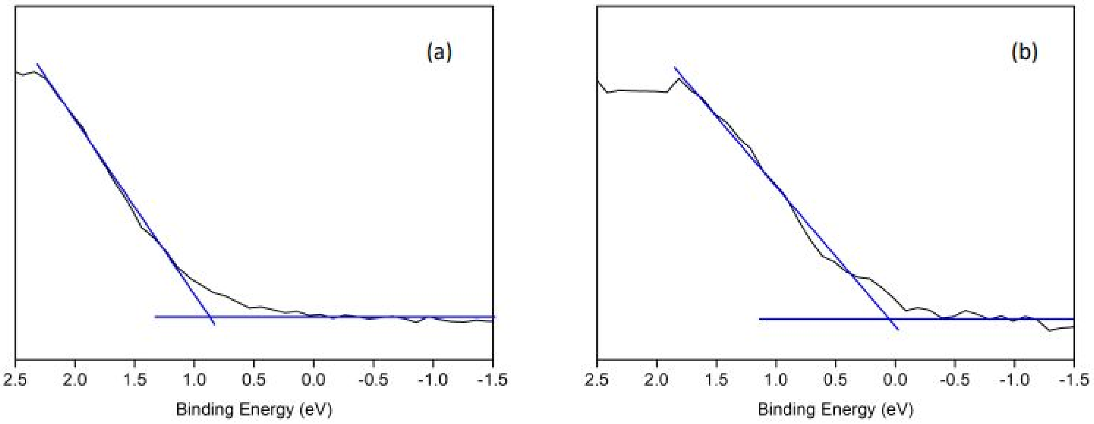

Fig. S10. (a) and (b) show the VBM of pristine and 2:1 Ga doped WS_2 samples, whose Ef are 1.6 eV and 1.1 eV relatively. The obvious shift toward lower binding energy indicates a p-type doping due to the Ga incorporation.

References

(1)Butanovs E.; Dipane L.; Zolotarjovs A.; Vlassov S.; Polyakov B., Preparation of functional Ga_2S_3 and Ga_2Se_3 shells around Ga_2O_3 nanowires via sulfurization or selenization, *Optical Materials* **2022***, 131*, 112675. https://doi.org/10.1016/j.optmat.2022.112675

(2)Gu, Y.;Serna, M. I.; Mohan S.; Lodoño-Calderon A.; Ahmed, T.; Huang Y.; Lee, J.; Walia S.; Pettes, M/ T. , Liechti K. M.; Akinwande D., Sulfurization Engineering of One-Step Low-Temperature MoS_2 and WS_2 Thin Films for Memristor Device Applications *Advanced Electronics Materials* **2022***, 8 (2)* 2100515. https://doi.org/10.1002/aelm.202100515

(3)Kresse, G.; Furthmüller, J. Efficient Iterative Schemes Forab Initiototal-Energy Calculations Using a Plane-Wave Basis Set. *Physical Review B* **1996**, *54* (16), 11169–11186. https://doi.org/10.1103/physrevb.54.11169.

(4)Tkatchenko, A.; Scheffler, M. Accurate Molecular van Der Waals Interactions from Ground-State Electron Density and Free-Atom Reference Data. *Physical Review Letters* **2009**, *102* (7). https://doi.org/10.1103/physrevlett.102.073005.

(5)Kieczka, D.; Thomas Robert Durrant; Milton, K.; Goh, J.; Bosman, M.; Shluger, A. L. Defects in WS_2 Monolayer Calculated with a Nonlocal Functional: Any Difference from GGA? *Electronic Structure* **2023**, *5* (2), 024001–024001. https://doi.org/10.1088/2516-1075/acc55d.
